# An Update on Career Trajectories and Professional Development Plans of MADURA Undergraduate Mentorship Program Alumni and Continuing Trainees

**DOI:** 10.24966/ggm-8662/100229

**Published:** 2024-09-17

**Authors:** S Thompson, B Marquez, E Fricovsky, DR Trinidad, A Molina, SD Edland

**Affiliations:** 1 UC San Diego Herbert Wertheim School of Public Health and Human Longevity Science, La Jolla, California, USA; 2 UC San Diego School of Pharmacy and Pharmaceutical Sciences, La Jolla, California, USA; 3 UC San Diego Department of Medicine, La Jolla, CA, USA; 4 UC San Diego San Diego Herbert Wertheim School of Public Health and Human Longevity Science, Department of Neurosciences, La Jolla, CA, USA

**Keywords:** ADAR, Aging, Alzheimer’s disease, Diversity, Measurement, Mentorship, MSTEM, Outcomes, Research, Undergraduate

## Abstract

By 2060, the U.S. population is projected to be older and more racially and ethnically diverse, and Underrepresented Minorities (URMs) are disproportionately affected by age-related conditions, including Alzheimer’s Disease (AD) and Alzheimer’s Disease Related Dementias (ADRD). There is a pressing need to improve diversity among aging and AD/ADRD researchers and clinicians, to better address needs of the current and future older adult population. Yet college students identifying as African American, Latinx, American Indian, first-generation, or sexual or gender minority individuals and individuals with disabilities are less likely to complete STEM bachelor’s degrees or subsequently utilize STEM degrees, than their non-Hispanic White counterparts. The Mentorship for Advancing Undergraduate Research on Aging (MADURA) Program supports undergraduate retention and success, graduate/medical school applications, and entry into aging and AD/ADRD research or clinical employment. This article reports on mentee graduate degree attainment and aging/AD/ADRD employment outcomes. It also details its cohort’s intermediate post-bachelor’s educational and career preparation activities, designed to prepare graduates to achieve longer term professional goals. Longitudinal research on mentees’ intermediate professional development activities and their utility is suggested, to improve understanding of trainees’ professional development pathways and optimally supportive mentorship program design.

## The Need for Improved Diversity in Aging, Alzheimer’s Disease and Alzheimer’s Disease-related Dementia Research and Clinical Professions

Many American racial and ethnic minority groups, including Hispanics/Latinos and Blacks/African Americans, are disproportionately affected with aging-related conditions, including Alzheimer’s disease and dementia [1,2]. Further, Vespa and colleagues cite projections that by 2060, non-Hispanic Whites will remain the single largest racial group but 32% of the U.S. population will be a race other than White. By 2060, those identifying from two or more races are predicted to be roughly 6%, Black/African American 15%, and Hispanics 28% [3]. Given this population shift, the vital need to reduce these health disparities becomes even more urgent. Developing and nurturing diverse talent in Medicine, Science, Technology, Engineering and Mathematics (MSTEM) skills is instrumental to improved prevention and treatment for all.

However, there is profound underrepresentation of people of color among researchers who study aging-related conditions and clinicians who serve these patients [4]. Educational disparities in MSTEM exacerbate this problem. A 2019 National Academies of Sciences Engineering and Medicine (NASEM) report indicates that individuals from underrepresented groups (those identifying as African American, Latinx, American Indian, first-generation, or sexual or gender minority individuals and individuals with disabilities) are less likely to be successfully integrated into academic and career MSTEM environments [5]. Progress has been made, with this NASEM *Science of Effective Mentorship in STEMM* report noting that 40+ years of federal investment in mentorship has resulted in increased STEM and medical baccalaureate and graduate degrees attained by these under-represented groups. Still, the report cites multiple studies indicating that Underrepresented Minority (URM) students who attain STEM bachelor’s degrees are 50% more likely to move on to graduate educational programs and/or fields outside of STEM, than those from well-represented groups. Therefore, in the quest to improve inclusion in aging, Alzheimer’s Disease (AD) and Alzheimer’s disease Related Dementias (ADRD) professions, we must assess post-graduation retention in key educational pathways and careers, in addition to bachelor’s degree attainment.

### Mentorship for aging and AD/ADRD careers

For practical reasons, mentorship programs typically have a relatively narrow focus appropriately attuned to their professional goals, institutional homes, expertise and resources, such as programs focused supporting graduates from a single educational major. Although not widely represented in the research literature, some reports of mentorship programs with a broader aging or AD/ADRD focus exist. Urena et al., [6] conducted a scoping review and noted a need for systematic evaluation standards for programs intended to increase diversity of researchers and practitioners in aging and AD.

Further, 2017 research specifically on neuroscience degrees determined that there has been tremendous overall growth in the number of neuroscience bachelor’s, master’s and doctoral degrees granted over the past 30 years, but that the proportion of URM degree attainment has only slightly increased. Ramos and colleagues note that “based on modest changes in the percentage of URM neuroscience graduates despite the substantial growth in total number of neuroscience graduates, we also predict that without major changes in the recruitment and/or retention of URM students, the percentages of graduates from among these racial/ethnic groups will continue grow but remain at low levels relative to White (non-Hispanic) graduates” [7]. One example of NIH work to improve representation in aging, AD and ADRD fields (including neurosciences) is the National Institute on Aging ADAR (Advancing Diversity in Aging Research) program. ADAR programs, including the UC San Diego Mentorship for Advancing Diversity in Undergraduate Research on Aging (MADURA) program, support a diverse pool of college undergraduates engaged in MSTEM studies and promote pursuit of careers in aging, AD or ADRD research and/or health services.

## MADURA mentorship program

The MADURA Program is an educational program at UC San Diego, supported by the National Institute on Aging. Its primary aim is to increase diversity among researchers and clinicians focused on aging and/or AD/ADRD. To this end, in the past four years MADURA has trained over 60 undergraduates from racial/ethnic groups historically underrepresented in MSTEM majors, and/or from disadvantaged backgrounds (per NIH criteria). Many trainees have participated over multiple years, an optimal scenario for deepening skills. While not the focus of this paper, program quality is germane to considering outcomes. Per our most recent alumni survey, effectiveness in supporting undergraduates from groups who are under-represented in MSTEM to enter careers in Aging, AD, or ADRD research or clinical careers was rated as “Excellent” by 84% of graduates and “Good” by another 13%.

## Background

In 2024, a paper summarizing MADURA alumni post-graduation activities was published in the International Journal of Aging and Human Development [8]. Thompson and colleagues reported on data from its first three years in existence, so data were limited. Nonetheless, some important themes emerged. Specifically, although many MADURA graduates intended to pursue post graduate education in furtherance of aging or AD/ADRD research or clinical careers, few entered educational programs immediately after bachelor’s degree attainment. The data revealed, however, that many were actively engaging in professional development activities designed to strengthen graduate or medical school applications. A key recommendation was that mentorship program directors collect outcome measures beyond graduate and medical school applications and acceptances, to ensure that intermediate progress towards these goals is also detected. This paper builds upon this prior research by adding data from a more recent 2023/2024 cohort, as well as a new set of alumni follow-up survey data collected in the second half of 2024.

### MADURA program structure and evaluation

Undergraduate trainees engage in 8 hours per week of paid aging or AD/ADRD-related research work in faculty labs. A wide variety of research studies are represented, including genetic and other wet lab studies, investigations employing animal models, human intervention studies and clinical trials, and epidemiological studies. Research work is overseen by faculty investigators but may also be directly supervised by postdoctoral researchers, graduate students or senior research staff. In addition to their 8 hours of work in labs, MADURA provides students with two hours per week of faculty led whole-cohort professional development training. These weekly whole cohort meetings are comprised of one hour of small group break-out discussions facilitated by MADURA faculty mentors, plus another hour of alternating skills trainings, research seminars, and occasional tours of research labs or rapport building social activities.

The MADURA program utilized much of the whole-cohort training time to promote advance planning of summer and post-graduation professional development activities. Application deadlines for such activities often occur in the last and first quarters of the calendar year, and prior evaluations indicated that new trainees were often unaware of these deadlines. Indeed, many did not expect application windows to open until late Spring, long past most actual due dates. Therefore, in November through February, MADURA hosted presenters who described many types of relevant summer and post-graduation training opportunities. Mentees were given program links, referrals, and skills trainings on searching opportunities and preparing strong applications. Perhaps most beneficial, visiting MADURA alums and other graduate and medical students shared their own positive experiences with internships, summer research grants, diversity supplements, etc. In order to evaluate effectiveness of these and other training activities, and to support continuous quality improvements, MADURA consistently surveys current trainees and graduates. Consistent with recommendations by Urena and colleagues, [6] MADURA graduate and alumni evaluations are intended to assist in informing development of standard mentorship program metrics, in addition to supporting MADURA outcome evaluation.

### 2023/2024 mentee cohort

In the 2023–24 academic year, MADURA provided paid training to a total of 35 students, its highest mentee census to date. Under the leadership of 22 faculty research investigators plus 22 additional supervisors, these undergraduates trained in 24 different aging/AD/ADRD research projects at UC San Diego and the UC San Diego Shiley-Marcos Alzheimer’s Disease Research Center (ADRC). Consistent with its mission to increase diversity in aging/AD/ADRD careers, this 2023/2024 cohort was comprised of a large proportion of students of color, who were the first in their family to attend college and/or met NIH disadvantaged criteria. Of the 35 trainees who participated as MADURA mentees during school year plus another three finishing up work over the prior summer, 47% (18) reported ethnicity as Hispanic/Latinx, 8% (3) American Indian/Alaska Native, 3% (1) Black or African American, 24% (9) non-Chinese Asian, and 24% (9) multi-racial and 23% (8) non-Hispanic White. Another 8 (23%) selected “unknown” or chose not to report race. Two trainees (5%) reported disabilities and 68% (26) came from disadvantaged backgrounds per NIH criteria.

The MADURA program celebrated 22 graduates at the end of this academic year, in June of 2024. Majors of the MADURA graduating seniors were: 6 Cognitive &/or Behavioral Neuroscience, 3 Neurobiology, 5 Human Biology, 1 General Biology, 1 Biochemistry, 1 Bioinformatics, 2 Clinical Psychology and 3 Public Health. Four had minors in Global Health or Ethnic Studies, two had Cognitive Science minors and another two had Psychology minors. These and all MADURA trainees since program inception have attained bachelor’s degrees, with the exception of 11 current mentees whose studies and MADURA participation will continue in the 2024/2025 academic year.

### 2023/2024 cohort career ambitions and preparation survey

All 38 mentees who had actively participated in MADURA during the summer of 2023 or the 2023/2024 academic year were invited to take this online survey, whether graduating or continuing with MADURA in the next academic year. Thirty-one students (82%) completed surveys, which queried about ultimate career aspirations and short-term (Summer 2024) educational and career development actions. As expected, our cohort contained a mix of those with clinical versus research ambitions. These are listed in order of prevalence, in table 1, below.

As hoped, at the time of the survey (May 2024), even continuing trainees had a variety of professional development and educational activities arranged for the summer months or already underway. Recent graduates were likewise strategic in pursuing strategic experience, as outlined in table 2.

### Alumni post graduation survey data sources and demographics

From December 2023 through mid-2024, we collected alumni follow-up data from 38 of 42 former MADURA trainees (90% response rate). The remaining students have been lost to follow-up. The Alumni survey was offered online and respondents were offered a completion incentive of a $50 Amazon gift card. The survey prompted for feedback on current professional and educational activities, as well as future plans. Given seasonal windows for applications to training and educational programs, it was assumed that alumni might need up to six months to implement some longer-term professional development plans. As with the 2023 Alumni dataset, all respondents had been separated from the program for six months or greater.

### Results from the alumni post graduation survey

Four respondents had graduated in 2024 (more than six months prior), 19 in 2023, 13 in 2022 and 2 in 2021. As related to program outcomes, a total of 13 respondents (34%) report already working in a research job related to aging/AD/ADRD while another 13 (34%) report working in a clinical job related to aging/AD/ADRD. Even though the majority of trainees graduated 2023 or later, eight trainees (21%) had been accepted or were already participating in graduate programs related to research, while another five (13%) were actively applying. Of those specifically with medical school ambitions: three (8%) had been accepted while another five (13%) were actively applying.

Importantly, a vast majority do not consider their education complete; 95% (36) report that they are continuing education in graduate/medical programs or preparing to do so. Most (74%) of our students were taking gap time “with plans to resume education or training.” Table 3 gives a comprehensive picture of collective alumni status, with comparisons of net percentage of each activity reported in 2023 and 2024.

### Professional development intentions

It is instructive to note career development activities that our mentees rule out, as well as those they pursue. We may then consider whether we might have omitted trainings on important strategies, and/or spent training time on some strategies that are not beneficial to most trainees. A substantial reduction in professional activity rule-outs occurred from 2023 to 2024 (Table 3, column 4). In 2024, more trainees saw potential relevance and planned to pursue additional research work, clinical/health service work, graduate school applications (to master’s or PhD programs), medical school applications, postbaccalaureate program participation, and focused GRE or MCAT preparation. Of these, intentions to apply to graduate school experienced the greatest gain, up 41% from 2023, while GRE/MCAT preparation gained 19% and engaging in clinical or health services work gained 18%.

### Current engagements

The survey results highlight increases in the percentage of trainees who were actively engaged or had received official acceptance into research jobs (up 10%), clinical or health service jobs (up 8%), short term internships or training programs (up 10%), longer term internships (up 11%) and medical school (up 1%). There was an increase of 8% in the proportion of students actively studying for the GRE or MCAT exam. The largest reduction in endorsed activities was a decrease of 18% in current paid work designed to strengthen medical or graduate program applications. However, there was 14% increase in alumni who were actively applying for these types of jobs.

### Longer-term plans

There were increases in the proportion of former mentees with 1–2 year plans to apply to graduate programs (up 18%) and medical school (up 13%). Not surprisingly, there were corresponding increases in students planning to engage in GRE or MCAT studies in the coming 1–2 years, as well (up 9%).

## Discussion

The MADURA Program provides training, referrals and resources in support of all strategies listed on the surveys. All are considered to be potentially helpful strategies on the path to careers in aging and AD/ADRD research or clinical careers. However, roughly half of the cohort endorses medical or health career ambitions while another half targets careers in research. A few also consider blending the two, as in the case of MD/MPH or clinician researcher careers. Given differing end goals, not all listed activities are of similar value for each trainee. Each mentee is supported in evaluating the merits of each professional development action, relative to their own career ambitions, accomplishments and current skill set.

Overall, the results of both surveys indicate substantial use of professional development activities encouraged by the MADURA program. Every single queried skill received some endorsement as being completed or pursued, by substantial numbers of trainees. Over two-thirds of 2024 respondents were underway or planning graduate (Master’s or PhD) studies, and another third were underway or planning medical or graduate health program studies. Almost all trainees, therefore, aim to continue their education beyond their bachelor’s degrees. Given the need for aging and AD/ADRD professionals from under-represented, first generation and disadvantaged backgrounds, this is highly encouraging. Also noteworthy is the benefit of alumni from these backgrounds who are already engaged in aging, AD or ADRD research or clinical employment. A remarkable 76% (29) of the 2024 respondents reported employment in these research or clinical settings. Even if an interim step before additional education, the work of these alumni already expands professional representation in aging, AD/ADRD research and clinical care settings. As such, these alumni are already fulfilling a key aim of the MADURA Program.

As expected from our direct experience with these highly motivated MADURA mentees, few deem themselves ready to submit competitive graduate or medical school applications immediately upon graduation. As concluded from the previous career trajectories paper, [8] the 2024 results again suggest that the majority of mentees who aspire to further education are taking strategic interim steps after obtaining a bachelor’s degree, but prior to submitting graduate or medical school applications. It is highly encouraging that so many are taking active steps to strengthen skill sets and application packages, as demonstrated by the gains in short term internship participation (up 10%), long term internship participation (up 12%), and GRE or MCAT study (up 8%). This more recent data again suggest that longitudinal follow-up of program graduates needed, as career trajectories continue to evolve over a course of years for many graduates. Expanding knowledge of mentee strategies for strengthening C.V.s could help mentorship programs to better support the professional development strategies chosen by imminent and recent graduates.

## Limitations and Future Directions

Given the restricted MADURA cohort sizes, all survey sample sizes are relatively small. Although percentages are calculated to allow some comparison from year to year, the frequencies of responses are also provided, to allow the reader to gauge the actual prevalence of a given response. Also, response bias cannot be ruled out. Finally, participant identities were anonymous in the earlier 2023 survey; therefore, an individual’s response patterns could not be compared over time. The ability to make inferences about the educational and career progression of given individuals is therefore limited. The 2024 and future Alumni surveys include individual identifiers, allowing for within participants comparisons at future time points. We will continue to solicit Alumni status updates as long as the MADURA Mentorship Program remains in effect and will report on professional development trends that emerge over time. To advance the fields of MSTEM and aging/AD/ADRD mentorship, we again encourage programs to not only publish on post-graduation outcomes such as educational program acceptances, but also on strategic intermediate actions likely to advance graduates on their educational or professional development trajectories.

## Figures and Tables

**Table 1:** Career ambitions: MADURA 2023/2024 Cohort (n=31).

Professional Objective	Career Type	Percent	Frequency
**Health Care – Physician or Nurse**	Clinical	68% Physician 3% Nurse	21 1
**Clinical Psychologist or Neuropsychologist**	Clinical	10%	3
**Research Scientist**	Research	10%	3
**Product Designer**	Research/Industry	3%	1
**Pharmaceutical Drug Discovery**	Research	3%	1
**Biostatistician**	Research	3%	1

**Table 2:** Short-term career preparation activities (n=31).

Summer Educational and Professional Development:	Percent	Frequency
**Clinical or Research Volunteer or Internship Position**	42%	13
**non-MADURA Paid Research or Clinical Work**	35%	11
**Taking One or More Courses**	23%	7
**MCAT/GRE Preparation (formal program or self-directed)**	23%	7
**Additional Career-enhancing Certifications (EMT, CNA…)**	19%	6
**Medical Postbacc Program**	3%	1
**Other: “Mental Health Break” (time off from education/work)**	3%	1

Note: Responses are not mutually exclusive.

**Table 3:** MADURA alumni educational and professional development activities, 2023 and 2024.

MADURA Alumni Activities	Actively doing now, or officially accepted	In Process with applications	Planed within 1–2 years	Not planned or N/A
Notes: Students may engage in multiple activities simultaneously. Due to rounding error, some items may sum to > or < 100%.				
**Paid work experience designed to strengthen graduate or medical school applications (not just for income)**				
2024 (n=38)	53% (20)	21% (8)	8% (3)	18% (7)
2023 (n=14)	71% (10)	7% (1)	7% (1)	14% (2)
Net Change (Percentage)	− 18%	+ 14%	+ 1%	+ 4%
**RESEARCH Work in a job related to Aging, Alzheimer’s Disease, ADRD**				
2024 (n=38)	39% (15)	5% (2)	8% (3)	47% (18)
2023 (n=14)	29% (4)	7% (1)	7% (1)	57% (8)
Net Change (Percentage)	+ 10%	− 2%	+ 1%	− 10%
**CLINICAL or HEALTH SERVICE WORK related to Aging, Alzheimer’s Disease, ADRD**				
2024 (n=38)	37% (14)	16% (6)	8% (3)	39% (15)
2023 (n=14)	29% (4)	0% (0)	14% (2)	57% (8)
Net Change (Percentage)	+ 8%	+ 16%	− 6%	− 18%
**Graduate school applications (master’s or PhD)**				
2024 (n=38)	26% (10)	13% (5)	32% (12)	29% (11)
2023 (n=14)	36% (5)	0% (0)	14% (2)	50% (7)
Net Change (Percentage)	− 10%	+ 13%	+ 18%	− 21%
**Medical School applications**				
2024 (n=38)	8% (3)	13% (5)	13% (5)	66% (25)
2023 (n=14)	7% (1)	21% (3)	0% (0)	71% (10)
Net Change (Percentage)	+ 1%	− 8%	+ 13%	− 5%
**Short-term (3 month or less) Internships or Summer training programs**				
2024 (n=38)	24% (9)	11% (4)	3% (1)	63% (24)
2023 (n=14)	14% (2)	14% (2)	0% (0)	71% (10)
Net Change (Percentage)	+ 10%	− 3%	+ 3%	− 8%
**Longer-term (3+ month) internship programs**				
2024 (n=38)	18% (7)	5% (2)	5% (2)	71% (27)
2023 (n=14)	7% (1)	21% (3)	0% (0)	71% (10)
Net Change (Percentage)	+ 11%	− 16%	+ 5%	0 %
**Post-baccalaureate program**				
2024 (n=38)	18% (7)	5% (2)	5% (2)	71% (27)
2023 (n=14)	21% (3)	0% (0)	0% (0)	79% (11)
Net Change (Percentage)	− 3%	+ 5%	+ 5%	− 8%
**Saving money for additional education**				
2024 (n=38)	66% (25)	5% (2)	8% (3)	21% (8)
2023 (n=14)	71% (10)	0% (0)	14% (2)	14% (2)
Net Change (Percentage)	− 5%	+ 5%	− 6%	+ 7%
**Studying to strengthen additional education applications (GRE or MCAT prep, for example)**				
2024 (n=38)	37% (14)	3% (1)	16% (6)	45% (17)
2023 (n=14)	29% (4)	0% (0)	7% (1)	64% (9)
Net Change (Percentage)	+ 8%	+ 3%	+ 9%	− 19%
